# The decline of local wisdom in managing the Wain River protected forest near Indonesia’s new capital city buffer zone

**DOI:** 10.1371/journal.pone.0333008

**Published:** 2025-09-25

**Authors:** I. Made Geria, Retno Handini, Emi Purwanti, Ni Putu Eka Juliawati, Unggul Prasetyo Wibowo, I. Gusti Komang Dana Arsana, I. Wayan Suparta, Truman Simanjuntak, Harry Octavianus Sofian, Titi Surti Nastiti, Gede Adi Wiguna Sudiartha, Anggi Putri Kurniadi

**Affiliations:** 1 Research Center for Environmental Archaeology, Maritime Archaeology and Cultural Sustainability, National Research and Innovation Agency, Republic of Indonesia; 2 Faculty of Forestry, Mulawarman University, Republic of Indonesia; 3 Geological Museum, Ministry of Energy and Mineral Resources, Republic of Indonesia; 4 Research Center for Food Crops, National Research and Innovation Agency, Republic of Indonesia; 5 Civil Engineering Department, Bali State Polytechnic, Bali, Republic of Indonesia; 6 Center for Prehistory and Austronesian Studies, Jakarta, Republic of Indonesia; 7 Research Center for Archaeometry, National Research and Innovation Agency, Republic of Indonesia,; 8 Research Center for Prehistoric Archaeology and History, National Research and Innovation Agency, Republic of Indonesia,; 9 Environmental Engineering Study Program, Faculty of Engineering, Udayana University, Bali, Republic of Indonesia; 10 Research Center for Macroeconomics and Finance, National Research and Innovation Agency, Republic of Indonesia; Universitas Airlangga, INDONESIA

## Abstract

The Wain River protected forest serves not only as a watershed but also holds a critical role in sustaining hydrological functions. The Sultan of Kutai Kertanegara Sultanate initiated ecological engineering efforts in 1934 by designating this area as a protected forest. However, rapid urbanization has led to a decline in local wisdom, posing a threat and intensifying pressure on the Wain River protected forest. The practices of local wisdom applied by the community within the Wain River protected forest area significantly impact the forest’s sustainability. Despite their diminishing influence, they still uphold ancestral guidance in forest conservation. Wain River is also a buffer zone forest for the new capital city of Indonesia at Penajam Paser Utara. Utilizing the Multidimensional Scaling (MDS) method, it was determined that the role of local wisdom in managing the Wain River protected forest falls under a category of weak sustainability, scoring 68.034 percent. Major influencing factors include land-use conversion and economic concerns, with the economic dimension scoring a sustainability rate of 62.83 percent. To foster the traditional agricultural economy, efforts are needed to maximize the utilization of Community Forests (CF) and capitalize on environmental services while ensuring the preservation of the Wain River protected forest.

## 1. Introduction

Local wisdom and social engagement are crucial for forest and water conservation [1]. Their loss threatens ecosystems, hinders resource management, and exacerbates conservation pressures [2,3]. The decline of local wisdom not only affects resource management but also disrupts overall ecosystem balance. Water, as a vital element, influences ecosystem stability [4].

The Wain River Protected Forest serves as a case study on the decline of local wisdom, partly due to the influx of urban communities unfamiliar with local forest management practices. This forest functions not only as a watershed (DAS) but also plays a crucial role in maintaining the hydrological ecosystem. Initially referred to as “Forest Cover,” it was officially designated as a protected forest by Sultan Kutai Kertanegara in 1934 through Royal Government Decree No. 48/23-ZB-1934 [5].

The Wain River is located at coordinates 116º 47’ – 116º 55’ East Longitude and 01º 02’ – 01º 10’ South Latitude. This area comprises the Wain River Protected Forest, which consists of primary and secondary forests that replaced those burned during the 1997/1998 fires, maintaining high biodiversity of flora and fauna [6]. Two rivers, the Wain River and the Bugis River, flow through this area, with their water stored in reservoirs to meet the needs of the local community.

Between 2002 and 2007, the Wain River area’s population increased by 35.58%, raising demand for space and environmental pressure [5]. Since 2017, the Forestry Office of East Kalimantan, with the Pro Natura Foundation, has managed the Wain River protected forest as a buffer zone for Indonesia’s capital. The area’s residents are mainly Paser ethnic, with 3,227 farmers living in Karang Joang Sub-District, mostly within the buffer zone. In 1972, some were relocated during the Wain reservoir’s expansion [7].

Ethnic groups such as the Javanese and Bugis began migrating to this region, bringing their ancestral wisdom and adapting to the new environment. Their approach is analyzed through Traditional Ecological Knowledge (TEK) [8,9], a collaborative concept that promotes shared learning in natural resource management. TEK encompasses knowledge, practices, and beliefs that are culturally inherited and adaptively evolve over time and across groups [10–12].

Migration patterns, particularly the influx of non-local ethnic groups like the Javanese and Bugis, can erode indigenous knowledge and practices, especially in natural resource management. These migrants bring different cultural beliefs, leading to value conflicts and the dilution of traditional customs. Poor adaptation of TEK to new environments has resulted in inefficiencies in resource management and the erosion of local governance structures. This cultural shift has hindered forest conservation efforts, causing biodiversity loss, altered land use, and imbalanced ecosystems. Understanding how migration and cultural changes impact local wisdom is crucial for sustainable forest management, especially in areas like the Wain River Protected Forest.

The local community has long depended on the forest, with native species being essential for ecosystem services, ecological balance, and biodiversity [13]. Recorded data shows that between 139 and 214 types of forest products are used for food, medicine, construction, income, and cultural practices. The dependence on the forest varies by generation and community group, with indigenous (Dayak) and migrant communities having different needs and uses for forest products [5]. These needs evolve due to cultural and economic changes, as well as land use changes that impact forest sustainability.

The forest functions not only as an economic resource but also as a social and cultural one [14]. For migrants around the Wain River Protected Forest, the forest is primarily utilized for socio-economic needs, while cultural diversity accelerates the decline of local culture and the Dayak understanding of forest conservation. Global studies show that the accelerated decline of TEK significantly impacts resource management and biodiversity [15–18].

With the increasing dependence of migrants on forests for socio-economic needs, this cultural shift has also accelerated the decline of local wisdom in forest management. This has implications for changes in perspectives and conservation practices, which were previously based on traditional knowledge. In this context, various studies highlight the importance of local wisdom in sustainable ecosystem management, particularly in fostering reciprocal relationships between humans and nature.

Abas et al. [2] identify that the loss of local wisdom exacerbates ecosystem decline and hinders the effective management of natural resources. However, a key gap in the research is the lack of knowledge regarding the specific impact of this cultural shift on forest conservation in certain areas, such as Sungai Wain, which has a long history of indigenous management.

This cultural shift is driven by the influx of ethnic groups and migration from other regions, as observed in Wain. Research on TEK provides insights into how traditional knowledge is managed and adapted to environmental conditions. However, its implementation often encounters challenges due to the adoption of new knowledge and lifestyle adaptations by migrants. Rujehan et al. [5] show that the increasing complexity of interactions between local cultures and migration leads to the erosion of traditional values in resource management, particularly in areas experiencing high migration pressure.

Additionally, although some studies indicate that local wisdom can contribute to ecosystem sustainability [18], there remains a significant knowledge gap regarding the factors driving the loss of local wisdom in the context of protected forests, such as in Wain. Previous research has primarily focused on conservation without adequately considering the social and cultural dynamics that directly influence forest management practices.

The phenomenon of local knowledge decline occurring in the Wain River Protected Forest reflects socio-ecological dynamics that are also unfolding in many parts of the world. Although the geographical context of this study is based in Indonesia, the research addresses global issues related to the erosion of traditional values due to migration, modernization, and land use change. Cross-country studies have shown that local communities and their traditional knowledge are increasingly marginalized in development processes, especially in buffer zones surrounding major cities or new capital areas [16]. In this context, the Wain River Protected Forest—now designated as a buffer zone for Indonesia’s new capital—offers a unique case that can enhance global understanding of how urbanization pressures accelerate the decline of traditional ecological knowledge. Therefore, this study is not only relevant to local readers but also to the global scientific community seeking strategies for conservation rooted in local wisdom in regions impacted by large-scale development. This perspective positions the study as a valuable contribution to the global discourse on integrating traditional knowledge into sustainable natural resource management.

In this regard, addressing the research gap on local wisdom is crucial to gaining a deeper understanding of how local traditions and social engagement can serve as effective tools in sustainable forest management. For example, understanding the ecological values embedded in indigenous rituals or local community practices can provide solutions for conservation efforts that are more inclusive and adaptable to the social changes occurring in the region.

This study reveals local wisdom in the sustainable management of the Wain River Protected Forest. The indigenous people of Kalimantan have developed effective methods to address forest decline, with sustainability relying on natural, economic, and cultural capital [15,19,20]. As part of the “ethnosphere,” local wisdom reflects human intuition in caring for the Earth. Although they make up only five percent of the global population, indigenous communities manage 11 percent of the world’s forests and protect 80 percent of biodiversity and 85 percent of global protected areas [21].

This article discusses the role of local wisdom in the management of the Wain River Protected Forest, the factors contributing to its decline, and the impact on sustainability. The study identifies the causes of the loss of local values in order to encourage more active community participation in forest conservation. The findings are expected to provide solutions and serve as a reference for conservation efforts to support the sustainability of the new national capital.

## 2. Materials and methods

### 2.1 Study area

This research was conducted in the Wain River Protected Forest (Hutan Lindung Sungai Wain) located in East Kalimantan, Indonesia, at coordinates 116º 47’ – 116º 55’ E and 01º 02’ – 01º 10’ S. The area comprises both primary and secondary forest, the latter having regenerated after a major fire in 1997–1998. The Wain and Bugis Rivers flow through this forest, with water from these rivers stored in reservoirs to meet the needs of the surrounding communities.

The forest is rich in biodiversity, including endemic flora and fauna. The surrounding population includes ethnic groups such as Paser, Bugis, Javanese, and Dayak communities, who have historically lived in and utilized the forest using traditional knowledge. Social and cultural dynamics in the area reflect interactions between indigenous traditions and migrant communities, influencing how natural resources are managed. As a buffer zone to Indonesia’s new capital city, the area offers a unique context to study how large-scale development pressures impact traditional ecological knowledge. A series of maps—starting with a world map, followed by maps of Indonesia, East Kalimantan, and finally the study site—illustrate the research location.

### 2.2 Data collection

This study employed a mixed-methods approach, integrating qualitative and quantitative techniques to assess the sustainability of forest management based on local wisdom. Data collection took place from May 26 to June 3, 2023. All respondents and informants participated voluntarily after receiving an explanation of the study’s objectives and provided informed consent. Their identities remain confidential, and all data were anonymized in compliance with standard research ethics. The Ethics Commission for Social and Humanities Research of the National Research and Innovation Agency has granted ethical clearance (Decision No: 320/KE.01/SK/05/2023) for this study.

Qualitative data were collected through in-depth interviews, focus group discussions (FGDs), and direct observation. Group interviews provided broader perspectives, while FGDs helped to gain deeper insights into natural resource management practices. Direct observations were conducted to examine community activities, ecosystem conditions, and the actual implementation of forest policies.

Quantitative data were collected from 90 respondents—nearly three times the recommended minimum for non-probability sampling in unknown population sizes. Participants included local residents who utilize forest resources and environmental services. Data were gathered through questionnaires, interviews, document reviews, and observations in Karang Joang Village. Observed variables included community activities such as harvesting firewood, rattan, bamboo, honeybee products, and other forest resources, as well as institutional roles in land use and forest protection. Respondents demonstrated ecological awareness and a strong commitment to sustainability for future generations.

#### Inclusion or exclusion criteria for 90 respondents.

The criteria for inclusion of respondents in this study were as follows:

- Age: Respondents must be at least 18 years old to ensure that they have a mature understanding of forest management.
- Length of Residence: Respondents who have lived for at least 5 years in the vicinity of the Sungai Wain Protected Forest, so that they are expected to have sufficient experience and involvement in interactions with the forest.
- Involvement: Respondents who are directly or indirectly involved in activities related to forest management, such as farmers, traditional leaders, local communities, or parties affiliated with conservation-related institutions.

These criteria are used to ensure that the data obtained comes from individuals who are relevant and have direct experience with natural resource management in the Sungai Wain Protected Forest.

#### Justification for selecting 90 respondents.

A non-probability purposive sampling approach was used in this study to select respondents based on specific criteria as mentioned above. The number of 90 respondents was determined based on the need to obtain representative data, taking into account the limitations of research resources.

The sample size was calculated using Slovin’s Formula, namely:


$$\text{n}\;=\;\text{N1}\;+\;\text{N}(\text{e2})\text{n}\;=\;\backslash \text{frac}\{\text{N}\}\{\text{1}\;+\;\text{N}({\text{e}}^{\wedge }\text{2})\}\text{n}\;=\;\text{1}\;+\;\text{N}(\text{e2})\text{N}$$

Assuming a population of 120 people (the community relevant to forest management) and a margin of error of 10%, the following is obtained:


$$\text{n}\;=\;\text{1201}\;+\;\text{120}(\text{0}.\text{12})=\text{90n}\;=\;\backslash \text{frac}\{\text{120}\}\{\text{1}\;+\;\text{120}(\text{0}.{\text{1}}^{\wedge }\text{2})\}\;=\text{ 90n}\;=\;\text{1}\;+\;\text{120}(\text{0}.\text{12})\text{120}\;=\;\text{90}$$

Power analysis was not explicitly conducted because the primary objective was to obtain relevant qualitative and quantitative data related to forest management, not to generalise to a broader population.

#### Flowchart of MDS analysis steps.

The following are the steps of the MDS analysis that have been carried out, which will be presented in the form of a flowchart:

1. Data Collection: Sustainability attribute data was collected from questionnaires and interviews.
2. Attribute Assessment: Each attribute was scored by respondents using a Likert scale.
3. Data Normalisation: Attribute values are normalised using the formula:

Yij=Xij−XminXmax−XminYij=\frac{Xij – Xmin}{Xmax Xmin}Yij=Xmax−XminXij−Xmin

1. Attribute Weighting: Weights are assigned based on the importance of attributes using the pairwise comparison technique.
2. Calculation of Sustainability Index: The index is calculated for each dimension (economic, socio-cultural, environmental, etc.).
3. Classification of Sustainability Status: The status is classified into weak, moderate, or strong categories based on the index value.

#### Attribute selection method.

The selection of sustainability indicators and attributes was carried out through:

- Literature Review: Referring to previous studies on local wisdom-based forest management [18,22].
- Focus Group Discussion (FGD): Involving local community leaders, academics, and environmental practitioners to identify specific attributes relevant to the local context.
- Expert Panel: Discussion with forestry experts to validate the proposed attributes.

This approach ensures that the attributes used in the analysis are relevant, valid, and contextual to the research location.

#### Numerical example of standardisation formula application.

An example of standardisation formula application will be included in the revised manuscript. For example:

If the economic attribute value for a particular respondent is 40, with a minimum value of 30 and a maximum value of 70, then the normalised value is calculated as follows:


Yij=40−3070−30=1040=0.25Yij = \frac{40 − 30}{70 − 30} = \frac{10}{40} = 0.25Yij=70−3040−30=4010=0.25

Numerical examples such as this will clarify how the analysis works for reproducing research.

#### Minimising researcher bias in qualitative methods.

To minimise researcher bias, the following steps were implemented:

- Data Triangulation: Data was obtained from various sources (interviews, focus group discussions, and document reviews) to ensure the validity of the information.
- Inter-Coder Reliability: Two independent researchers verified the results of the qualitative data coding.
- Reflective Journaling: Researchers recorded personal reflections during the research process to identify potential subjective bias.

These steps ensure that the research results are more objective and credible.

### 2.3 Data analysis

Sustainability assessment was conducted using MDS to evaluate the attributes of local wisdom-based management across six dimensions. The analysis process involved ordination using RAPFISH software [22] and the calculation of a sustainability index for both the general condition and each individual dimension. The assessment phase evaluated each attribute on an ordinal scale according to sustainability criteria in each dimension. This involved ordination analysis using the MDS method, index construction, and the determination of the sustainability status of local wisdom functions in management, covering both the general condition and each dimension [23]. The MDS pairwise comparison technique provided relative weights for the criteria, allowing for an in-depth evaluation of sustainability factors [24].

The MDS technique maps similar objects or points together, portraying them as adjacent points. Conversely, dissimilar objects or points are represented as distant points. The scores for each attribute form an X matrix (*n* × *p*), where *n* represents the number of regions with reference points and *p* is the number of attributes used. These scores are then standardized for each attribute, ensuring uniform weighting for each attribute and eliminating differences between measurement scales [24,25].

The following are the stages of MDS along with their general explanations:

a Determining dimensions and attributes based on the issues at hand.
b Weighting the data obtained from opinions and real conditions in the research area through pairwise comparison.
c Establishing primary references as either “good” or “bad” by scoring across all variables/attributes.
d Creating two additional main points: the “midpoint” representing both the bad and good points. These serve as vertical directional references (“up” and “down”).
e Establishing additional reference points known as “anchors” to assist in the ordination results.
f Creating ordination for all attributes within each dimension. The results of the ordination are visualized through horizontal and vertical axes. Through a rotation process, the positions of points can be visualized on the horizontal axis, assigning a sustainability index value of 0 percent (bad) to 100 percent (good).
g Standardizing score values for each attribute using the following formula:

$$X_{ik}sd=X_{ik}-X_{k}S_{k}$$

Where:

𝑋_𝑖𝑘_𝑠𝑑 = Standardized score value for each region (including its reference points) for *i* = 1, 2, ... *n*, for each attribute for *k* = 1, 2, ... *p*;

𝑋_𝑖𝑘_ = Initial score value for each region (including its reference points) for *i* = 1, 2, ... *n*, for each attribute for *k* = 1, 2, ... *p*;

𝑋_𝑘_ = Midpoint score value for each attribute for *k* = 1, 2, ... *p*;

𝑆_𝑘_ = Standard deviation of score values for each attribute for *k* = 1, 2, ... *p*.

Field observations, analyses, and secondary data informed the scoring of each attribute, reflecting the sustainability of management dimensions. Scores ranged from “poor,” indicating the least favorable condition for sustainability, to “good,” representing the most favorable. Intermediate values were assigned based on the number of rankings for each attribute. The sustainability status of management functions in local wisdom was categorized into four levels based on index values [26,27]:

- **0 ≤ index ≤ 25**: Not sustainable
- **25 < index ≤ 50**: Less sustainable
- **50 < index ≤ 75**: Weakly sustainable
- **75 < index ≤ 100**: Sustainable

The sustainability index for local wisdom functions was further analyzed using sensitivity analysis to identify attributes most critical to sustainability. This was determined by evaluating changes in the Root Mean Square (RMS) ordination along the x-axis (sustainability scale). Larger RMS changes resulting from attribute removal indicated higher sensitivity, highlighting the attribute’s significance in shaping the sustainability of forest management functions through local wisdom.

Secondary data was obtained from the Wain River Protected Forest Management Agency (BPHLSW) and the Karang Joang Sub-district Office to explore policies and regulations related to forest management. A literature review also contributed to enriching this analysis.

## 3. Results

### 3.1 The role of TEK in the Wain River protected forest conservation

Indonesia’s Minister of Environment and Forestry Regulation on local wisdom in natural resource and environmental management underscores the need to reexamine ecological wisdom. Local wisdom is a vital cultural system, deeply embedded in a community’s way of life, and evolves to address the challenges of sustaining life and growth. Rooted in the values and circumstances of a society, it serves as a guiding framework for navigating life’s complexities and promoting sustainable development. This is evident among some members of the Karang Joang community, who, despite relocating, continue to honor their ancestral traditions. On specific occasions, they visit their ancestral village to pay respects at forefathers’ graves, seeking blessings for success in life and agriculture.

The traditional wisdom of the Wain River community highlights their enduring connection to the forest. Tombstone inscriptions from 1928 AD and 1351 Hijri (1930 AD) indicate their settlement predated the Kutai Kertanegara Sultanate’s designation of the area as protected. Initially coexisting harmoniously with the Sultanate’s policies, the community was relocated in 1975 during the construction of the Wain reservoir. This relocation acknowledged their inseparable bond with the forest and their cultural practices, aligning with sustainability principles recognized at the time.

The Sultanate’s efforts to protect the forest underscored the importance of hydrological cycles vital for the region’s rivers, which remain crucial to the local water supply, including Balikpapan’s Wain Reservoir. Community practices, such as sustainable harvesting of rattan, bamboo, nipa palm leaves, fruits, and honey, reflect a balance between utilization and conservation. These activities, guided by traditional norms and taboos (pamali), emphasize sustainability over exploitation. Local customs prohibit actions like excessive resource use, tree cutting, unauthorized forest entry, hunting, and harmful practices. Such beliefs and regulations have become ingrained in their social structure, fostering positive impacts on forest conservation. As noted by Prasetyo [28], in several cases of forest management by communities, it has been observed that the wisdom of the people in managing forests has resulted in positive impacts on forest conservation. Due to their high dependence on the forest, their patterns of utilization tend to prioritize sustainability.

TEK reflects communities’ historical practices in adapting to their environments through socio-cultural, technological, and agricultural activities. Spiritual beliefs often guide sustainability efforts, including the preservation of ecosystems and adherence to prohibitions (pamali). The Wain River community demonstrates environmentally conscious practices, such as resource-efficient technologies and sustainable agriculture, rooted in local knowledge. This knowledge safeguards ecological processes, biodiversity, and ecosystems, ensuring sustainable economic benefits. Recognized since the 1990s in ethnobiology, TEK underpins development in fields like medicine, agriculture, forestry, and conservation. It represents the knowledge, practices, and beliefs integral to communities’ sustainable livelihoods, as seen in the Wain River protected forest.

In recent times, extensive urbanization and changes in land ownership have led to land use conversions. At KM 11 and KM 13 in Karang Joang, factory buildings have emerged, exerting pressure on the buffer zones. This development’s impact affects the sustainability of local wisdom ingrained in the lives of farming communities. However, there are still local communities that honor the guidance of their ancestors, primarily among the Dayak Paser people. In the Wain River protected forest area, various forms of ecological engineering activities have been conducted, providing evidence that locally designed systems could be useful in managing ecological processes across different observable scales. The evidences are as follows:

(a) The initial evidence comes from the belief system, where one practice involves venerating sacred graves and implementing measures primarily aimed at preserving the forest. A tradition that endures among the local people involves visiting ancestral graves to seek safety for both the community residents and the forested area. The value derived from this wisdom lies in their ancestors’ lives and growth within the forest, understanding the ethics of adaptation and forest conservation. They understand how to sustainably utilize the environmental services provided by the forest. In addition, they are rooted in a deep-seated intimacy and concern for their homeland, integrated both materially and spiritually within their landscape. TEK represents rational and dependable knowledge developed over generations through an intimate relationship between indigenous people and their land [29]. The assumption that TEK constitutes fundamental knowledge often aligns with the notion that the community’s worldview elements intricately weave into their TEK system. Berkes defines TEK as “a body of knowledge, practices, and beliefs accumulated and evolved through adaptive processes, transmitted culturally across generations, concerning the relationships between living beings (including humans) and the environment” [12,30]. This definition situates TEK as a corpus of “knowledge, practices, and beliefs” inspired by specific worldviews and bioregions, interwoven into the community’s cultural tapestry.
(b) The second evidence lies in ecological engineering through policies, as issued by the Kutai Kertanegara Sultanate to safeguard the forest and ensure hydrological sustainability. Dam construction technology utilizes water resources while simultaneously preserving the forest as part of the hydrological cycle. This endeavor is believed to have considered the geological conditions of the region, particularly the presence of alluvial plains resulting from the breakdown of claystone, which usually forms waterproof surfaces, delaying water absorption into the soil. The existence of claystone complicates the infiltration of surface water into the ground. Theoretically, due to its impermeable nature, claystone makes it difficult for groundwater to be obtained and stored. The presence of impermeable layers on the soil surface in the Wain River protected forest is manifested in the form of natural ponds or reservoirs like the Wain River dam [31]. The policy initiated by the Sultan of Kutai Kertanegara Sultanate became a reference for the construction of the first Wain River reservoir, built by the colonial oil company, namely Bataafsche Petroleum Maatschappij (colloquially known as BPM) in 1947, covering an area of 0.7 hectares. Subsequently, in 1969, SHELL took over the reservoir’s operation. From 1972 to date, PERTAMINA has managed it, with a reservoir area of 3.1 hectares. Currently, PERTAMINA utilizes around 15,000 cubic meters of reservoir water per day, meeting about 26% of Balikpapan’s clean water needs. This sustainable water supply helps prevent floods and meets domestic, agricultural, and fishing water requirements for Balikpapan and surrounding areas, particularly for those in the buffer zone of the protected forest area. The Kutai Kertanegara Sultanate holds significance as a pioneer in safeguarding the Wain River Forest as a protected area. Ecological engineering efforts were focused on safeguarding the forest to preserve hydrology and ensure the river’s safety, which traverses the forest area (see Fig 1 in S1 Appendix).
(c) The third evidence involves sustaining the Wain River protected forest by utilizing forest services with a strategy that considers sustainability. For instance, thatch craftsmen use forest services in the forms of rattan, nipa palm leaves, and bamboo. They selectively harvest mature rattan from the forest, leaving sprouting shoots to encourage further growth. Similarly, they use mature nipa leaves to ensure continued growth. These craftsmen employ forest services derived from renewable forest elements (see Fig 2 in S1 Appendix and Table 1).
(d) The fourth evidence lies in the implementation of community forests in the buffer zones of the Wain River protected forest. The ecological aim is to economically benefit the community by utilizing sustainable agricultural land management. Several practices by farmers support policies aimed at conserving the protected forest area, such as terracing steep slopes to prevent landslides and cultivating protective plants. However, decline occurs in the community forests due to land conversion, thereby posing a threat to the safety of the protected forest. This situation also leads to the loss of certain local farming community wisdom in the buffer zones of the Wain River protected forest.

**Table 1:** roles of ecological wisdom in managing the Wain River Protected Forest.

Ecological Elements	Environmental Services/Utilization	Role of Ecological Wisdom
Management of protected forests	The forest cover was initially enforced during the Kutai Kertanegara Sultanate’s rule in 1934, aiming to protect water catchment areas, ensure forest safety, and secure water availability for the Balikpapan community.	Water management and preservation of the Wain and Bugis Rivers’ ecosystems
Development of a 3.1-hectare reservoir	Addressing the interests of PERTAMINA and the water requirements of the Balikpapan community	Ensuring sustainable water availability
Population relocation efforts	Guaranteeing clean water availability upstream	Riverbank conservation
Sacred burial sites	Acknowledging the ancestral ties of the Dayak Paser community residing in the forest	Forest preservation
Extensive community forests covering 1,400 hectares	Aiming to boost the economy of communities living in buffer zones	Community involvement in land utilization and forest conservation efforts
Spatial planning regulations	Ensuring forest safety and sustaining community livelihoods	Protection of forested areas
Sustainable tourism zones	Enhancing community well-being through environmental services	Utilization of environmental services, promoting forest area preservation, and nurturing an awareness and love for forests
Botanical gardens	Advancing knowledge development	Biodiversity conservation
Fishing areas	Ensuring community food security	Cultivation of freshwater fish
Involvement of PERTAMINA (Indonesia’s state-owned oil and gas corporation)	Utilizing water resources for the PERTAMINA refinery while focusing on forest preservation	PERTAMINA’s CSR engagement in forest preservation
Government and management bodies	Sustaining protected forests	Ensuring forest safety
Traditional organizations		
Rituals like *Belian* and *Ngarak Wani* (rituals involving honey hunting in the forest and customary land clearing)	Cultivating appreciation and understanding of nature	Spiritually protecting the Wain River Forest area

### 3.2. The sustainability status of TEK in managing the Wain River protected forest

Observing the decline that has occurred in local wisdom regarding the management of the Wain River protected forest, several influential factors have been identified as contributing to the loss of TEK components. These include local knowledge about forest management, traditional farming systems, and social institutions guiding resource management. Direct threats could arise due to excessive exploitation that disregards local values in utilizing natural resources, deviating from sustainable norms [18]. The contributing factors to the direct decline of TEK usually encompass cultural, economic, political, and institutional aspects, as evidenced in several case studies [32–35]. These factors are field-tested using the MDS method.

The MDS analysis results indicate that overall, the status of local wisdom in managing the Wain River protected forest falls within the weakly sustainable category with a score of 68.034 percent. This condition is influenced by several dimensions. The economic and infrastructure dimensions fall into the weak category, whereas the environmental, social-cultural, and legal-institutional dimensions fall into the strong sustainability category (see Fig 3 in S1 Appendix). The environmental dimension scores at 71.83 percent sustainability, the economic dimension at 62.83 percent sustainability, the social-cultural dimension at 68.52 percent sustainability, the infrastructure-technology dimension at 67.38 percent sustainability, and the legal-institutional dimension at 69.61 percent sustainability.

In managing the environmental services of the Wain River protected forest, particularly in the economic and infrastructure-technology dimensions, there is a need for enhancement. Several challenges exist in the realms of the economy as well as infrastructure and technology, such as the relatively low average income of the community compared to the regional minimum wage, the stability of agricultural product prices when utilizing the services of the Wain River protected forest, and the commitment of the regional government to establish the necessary infrastructure for utilizing services of the protected forest. Moreover, the suitability of technology and government aid in that area requires prioritized attention. While respondents provide satisfactory explanations regarding forest and water resource management, the benefits from these natural resources are not reaped by the residents who safeguard them. This disparity requires careful consideration and rectification to ensure that those preserving natural resources receive more substantial benefits, especially in the community’s economic sector.

### 3.3. Identification of influential attributes on sustainability dimensions

Leverage analysis results indicate that 10 attributes hold relatively significant influence over the sustainability value within each dimension. Two attributes are chosen for each dimension as pivotal factors with a sensitive impact on sustainability, which will be prioritized for improvement. The attributes significantly affecting the sustainability of local wisdom in managing the Wain River protected forest are as follows.

1) Employment absorption rate in utilizing environmental services of the Wain River protected forest and the community’s role in preserving local cultural values to safeguard the protected forest,
2) Rate of conflicts associated with the utilization and conservation of the Wain River protected forest,
3) Stability of agricultural product prices when utilizing environmental services of the Wain River protected forest,
4) Average community income relative to the regional minimum wage,
5) Rate of agricultural land conversion to non-agricultural use,
6) Government support in spatial planning protection within the area,
7) Clarity of the local community’s status within the organizational institution of the Wain River protected forest area,
8) Government commitment and firmness in regulating community forest permits through protected forest land rules to prevent easy conversion,
9) Government commitment to providing facilities and infrastructure, and
10) Appropriate technological usage to enhance the value of the Wain River protected forest.

The analysis of the social-cultural sustainability index shows a score of 68.52 percent (see Fig 3 in S1 Appendix). Even though people residing around the Wain River protected forest no longer follow the Dayak Paser belief system, they still uphold local traditions. Respecting ancestral graves and sanctifying the areas around these graves are seen as efforts toward conservation. Similarly, the belief in “*pamali*” (taboos), which involves customary prohibitions regarding the use of nature and the environment, holds significance in safeguarding natural resources for their protection and sustainability. Preserving traditional wisdom faces challenges and threats due to disparities in urban communities’ social and cultural conditions.

As shown in Fig 4 in S1 Appendix, there are three key attributes associated with socio-cultural sustainability: the labor absorption rate in utilizing the environmental services, the communities’ role in preserving local cultural values, and the level of conflicts related to the utilization and conservation of the Wain River protected forest which is identified as the most sensitive attribute, with a sensitivity score of 0.63 compared to other socio-cultural attributes. The resulting impacts of leveraging the aforementioned factors are exhibited in Table 2. The community’s role in preserving local cultural values to protect the Wain River protected forest is currently weak, as the environmental services provided by the forest do not adequately meet the community’s livelihood needs. Additionally, the low employment rate is due to suboptimal forest management practices. To address these issues, it is suggested to transform the area into a sustainable tourist destination, focusing on specialized tourism that emphasizes environmental conservation and local cultural wisdom. This approach aims to reduce conflicts related to the utilization of the protected forest.

**Table 2:** leverage factors influencing the decline of local ecological wisdom (traditional ecological knowledge).

Dimensions	Significant Influencing Attributes	Examples of Resulting Impacts
Social and Cultural Dimension	Employment absorption rate in agriculture and forestry sectors and the utilization of environmental services	Forest environmental services are underutilized for community welfare.
	Community’s role in preserving local cultural values to maintain the Wain River protected forest	Limited community involvement exists in preserving cultural values due to differing beliefs, yet they still value the existence of local wisdom.
	Level of conflicts related to the utilization and conservation of the protected forest	Timber theft occurs frequently, primarily by non-local individuals, while local communities are not maximally engaged in utilizing forest environmental services.
Economic Dimension	The community’s average income relative to the regional minimum wage	Artisans providing environmental services face extremely low income, selling a sheet of thatched roof (made from nipa palm leaves) for only 2,000 IDR.
	Stability of agricultural product prices in utilizing the environmental services of the Wain River protected forest	Communities market independently their products without government assistance.
Environmental Dimension	Rate of agricultural land conversion to non-agricultural use	The land is subdivided for housing in buffer zones.
	Government support in spatial planning protection within the area	Weak regulations lead to excessive urbanization beyond the area’s carrying capacity; numerous factory buildings are erected at KM 11.
Legal and Institutional Dimension	Commitment (agreement) of community organization members not to repurpose their fields	Solutions are lacking for local communities to economically benefit from utilizing environmental services and cultivated land under the forest management permits, thus preventing land repurposing.
	Member status within the institution or the entry-and-exit regulations of members in the community organization	Traditional organizations have disappeared, with residents excluded from management roles as regional authorities delegate protected forest management to third parties.
Technology and Infrastructure Dimension	Regional government commitment to providing infrastructure for the utilization of the environmental services of the Wain River protected forest	Efforts to develop this area into a specialized tourism destination to boost the local economy are lacking.
	Suitability of technology and government assistance in the Wain River protected forest	Eco-friendly technology and government support for assisting communities in utilizing environmental services are absent.

Despite the high sustainability score in the socio-cultural dimension, the analysis results of the sustainability index for the economic dimension indicate a lower score of 62.83 percent. Compared to the multidimensional outcomes, the economic dimension demonstrates a weak sustainability status. Economic stability significantly influences the preservation of local culture in managing the protected forest area. From the sensitivity analysis in Fig 4 in S1 Appendix, two major attributes pose significant influence on the economic dimension, namely the average income of the community relative to the regional minimum wage and the stability of agricultural product prices in utilizing the environmental services of the Wain River protected forest. The government has initiated efforts to improve community welfare through the allocation of community forest permits in the buffer zone of the Wain River protected forest. Ideally, communities granted permits should effectively manage their cultivated land to sustain their livelihoods while ensuring conservation and sustainability within the local area. However, field observations indicate a tendency among them to think pragmatically, aiming for benefits by repurposing land for non-agricultural uses.

This issue undoubtedly poses a threat to the continuity of the traditional values upheld by agricultural communities in environmental preservation. According to Ruifei Tang and Michael C. Gavin, the abandonment of traditional livelihoods due to the shift from traditional subsistence to non-traditional activities, such as industrial and trade pursuits, is a contributing factor [18,36]. Several underlying factors need attention as leverage points, including the necessity to increase prices for agricultural products and traditional industries. For instance, craftsmen making roofs from nipa palm leaves sell each piece for only 2,000 IDR, which is unsustainable. This discrepancy significantly impacts the average earnings of farmers, not aligning with the relative average income compared to the regional minimum wage. The comprehensive value of attributes within the economic dimension is depicted in Fig 4 in S1 Appendix.

The environmental dimension of the Wain River protected forest exhibited the highest sustainability index among all multidimensional outcomes, with a score of 71.82 percent. In this dimension, two sensitive attributes significantly impact sustainability: the conversion of agricultural land to non-agricultural use and government support in spatial planning and protection within the area. However, it is vital to note that these two sensitive attributes need attention due to the increasing land use conversion and inadequate government support in securing the area’s spatial planning. Field observations at KM 11 and KM 13 indicate industrial development encroaching into the buffer zone of the Wain River protected forest, with land being cleared for housing plots within this zone. Consequently, the forest is encroaching upon residential areas.

The sustainability index analysis for the legal-institutional dimension stands at 69.61 percent. Although this is lower than the environmental dimension, it still reflects a strong sustainability status when compared to the multidimensional outcomes. Overall, the legal and institutional dimensions appear robust. Nevertheless, as explained in leverage factors in Table 2, there is an undeniable loss or alteration in traditional social organization, customary norms, and traditional rules regarding the use and management of resources. Leverage attributes needing attention include the status of the local community within the organizational structure of the Wain River protected forest, particularly concerning their role in managing the forest buffer zone and their associated rights and obligations. Similarly, the government must demonstrate commitment and firmness in regulating the use of community forest permits through protected forest land rules to prevent easy land use conversion. This is significant given the decline of traditional regulations and belief systems (*Belian*) among indigenous communities historically responsible for protecting the forest.

The sustainability index analysis for the technology-infrastructure dimension is 67.38 percent. Compared to the multidimensional outcomes, this dimension displays a weak sustainability status. There is a loss of traditional access to natural resources. Two crucial factors influencing this weakness are the government’s low commitment to providing infrastructure and facilities and the lack of suitable technology applications. Currently, the efforts in infrastructure preparation are suboptimal and do not meet the local community’s needs. Therefore, the community should be aided with technology to enhance the value of the Wain River protected forest and receive training in sustainable environmental practices that do not harm the environment but add value to the local economy, aligning with proven local wisdom in nature conservation.

## 4. Conclusions

This study demonstrates that the Sungai Wain Protected Forest, designated as a protected area in 1934, continues to provide vital ecological services, particularly in maintaining hydrological stability and biodiversity. The sustainability analysis indicates that the role of local wisdom in forest management falls into the weakly sustainable category (68.03%). Strength lies in the social and legal dimensions, reflecting community awareness of ecological balance and regulatory frameworks that support conservation. However, weaknesses are evident in the economic and infrastructure dimensions, where unstable agricultural prices, low community income, and limited technological support remain significant barriers.

These findings highlight the erosion of TEK due to land-use conversion and socio-economic pressures in buffer zones. The decline in TEK undermines community involvement and long-term forest sustainability. Strengthening the integration of traditional practices with modern approaches is therefore essential to safeguard ecological functions while ensuring equitable benefits for local people.

Policy actions are needed to address these gaps. First, enhancing community skills and access to eco-friendly technologies can increase economic value without degrading ecosystems. Second, developing TEK-based ecotourism provides opportunities to diversify local livelihoods while promoting conservation. Third, revitalizing traditional institutions and enforcing stricter regulations on land conversion are critical to maintaining cultural integrity and ecological resilience.

In conclusion, the future of the Sungai Wain Protected Forest depends on balancing conservation with socio-economic development. By reinforcing traditional knowledge, improving economic incentives, and supporting sustainable infrastructure, this protected forest can continue to serve as a strategic ecological buffer for Indonesia’s new capital city while ensuring community well-being and long-term sustainability.

## Supporting information

S1 Data Data. (XLSX)

S1 Appendix Appendix. (DOCX)

## References

[1] Liu J, Ouyang Z, Yang W, Xu W, Li S, Pascual U. Evaluation of Ecosystem Service Policies From Biophysical and Social Perspectives: The Case of China. Encyclopedia of Biodiversity. Elsevier. 2024. p. 300–14. doi: 10.1016/b978-0-12-822562-2.00359-5

[2] Abas A, Aziz A, Awang A. A Systematic Review on the Local Wisdom of Indigenous People in Nature Conservation. Sustainability. 2022;14(6):3415. doi: 10.3390/su14063415

[3] Boros A, Gordos B, Tőzsér D. A bibliometric analysis-based literature review of the relationship between sustainable water management and green innovations in the agricultural sector. Heliyon. 2024;10(12):e33364. doi: 10.1016/j.heliyon.2024.e33364 39027460 PMC11254601

[4] Grizzetti B, Lanzanova D, Liquete C, Reynaud A, Cardoso AC. Assessing water ecosystem services for water resource management. Environmental Science & Policy. 2016;61:194–203. doi: 10.1016/j.envsci.2016.04.008

[5] Rujehan R, Hadriyanto D, Supriadi A. Economic motivation of farming practices and their potential impact on the forest ecosystem: A case study in Sungai Wain protection forest (SWPF), Indonesia. IJSF. 2012;:31–46.

[6] Rujehan. Model pemanfaatan dan strategi managemen hutan lindung studi pengelolaan hutan lindung sungai wain kalimantan timur. Kiswanto, Editor. Mulawarman University Press. 2017.

[7] Fredriksson GM, Kam de M. Strategic plan for the conservation of the Sungai Wain protection forest East Kalimantan. 1999.

[8] Chanthayod S, Zhang W, Chen J. People’s Perceptions of the Benefits of Natural Beekeeping and Its Positive Outcomes for Forest Conservation. Tropical Conservation Science. 2017;10. doi: 10.1177/1940082917697260

[9] Hayati SD, Qayim I, Raffiudin R, Ariyanti NS, Priawandiputra W, Miftahudin M. Traditional Knowledge of Plants for Sunggau Rafters on Three Forest Types for Conservation of Apis dorsata in Indonesia. Forests. 2024;15(4):657. doi: 10.3390/f15040657

[10] Berkes F. Sacred ecology: traditional ecological knowledge and resource management. Taylor and Francis. 2012.

[11] Berkes F, Folke C, Gadgil M. Traditional Ecological Knowledge, Biodiversity, Resilience and Sustainability. Ecology, Economy & Environment. Springer Netherlands. 1994. p. 269–87. doi: 10.1007/978-94-011-1006-8_15

[12] Gadgil M, Berkes F, Folke C. Indigenous Knowledge for Biodiversity Conservation. Human Environment. 1993;22(2).

[13] Mattea S, Monge JJ. Safeguarding iconic tree species, dependent ecosystems, and regional economies: A New Zealand perspective on controlling Kauri Dieback. Trees, Forests and People. 2025;19:100742. doi: 10.1016/j.tfp.2024.100742

[14] Frick J, Bauer N, von Lindern E, Hunziker M. What forest is in the light of people’s perceptions and values: socio-cultural forest monitoring in Switzerland. Geogr Helv. 2018;73(4):335–45. doi: 10.5194/gh-73-335-2018

[15] Alcorn JB, Toledo VM. Resilient resource management in Mexico’s forest ecosystems: the contribution of property rights. In: Berkes F, Folke C, Editors. Linking Social and Ecological Systems: Management Practices and Social Mechanisms for Building Resilience. Cambridge University Press. 1998. p. 216–49.

[16] Berkes F, Colding J, Folke C. Rediscovery of traditional ecological knowledge as adaptive management. Ecological Applications. 2000;10(5):1251–62. doi: 10.1890/1051-0761(2000)010[1251:roteka]2.0.co;2

[17] Martinez-Reyes J. Biocultural Diversity Conservation: A Global Sourcebook. EBL. 2012;3:61–2. doi: 10.14237/ebl.3.2012.46

[18] Tang R, Gavin MC. A classification of threats to traditional ecological knowledge and conservation responses. Conservation and Society. 2016;14(1):57–70.

[19] Hariram NP, Mekha KB, Suganthan V, Sudhakar K. Sustainalism: An Integrated Socio-Economic-Environmental Model to Address Sustainable Development and Sustainability. Sustainability. 2023;15(13):10682. doi: 10.3390/su151310682

[20] Mensah J. Sustainable development: Meaning, history, principles, pillars, and implications for human action: Literature review. Cogent Social Sciences. 2019;5(1). doi: 10.1080/23311886.2019.1653531

[21] Robbins J. Native Knowledge: What Ecologists Are Learning from Indigenous People. Yale School of the Environment. 2018.

[22] Kavanagh P. Rapid appraisal of fisheries (Rapfish) project: Rapfish software description (for Microsoft Excel). University of British Columbia: Fisheries Centre. 2001.

[23] Fauzi A. Teknik analisis keberlanjutan. PT Gramedia Pustaka Utama. 2019.

[24] Cox MAA, Cox TF. Multidimensional Scaling. Handbook of Data Visualization. Springer Berlin Heidelberg. 2008. p. 315–47. doi: 10.1007/978-3-540-33037-0_14

[25] Nurmalina R. Analisis indek s dan status keberlanjutan system ketersediaan beras di beberapa wilayah Indonesia. Jurnal Argo Ekonomi. 2008;26(1):47–79.

[26] Susilo S. Keberlanjutan Pembangunan Pulau pulau kecil: Studi kasus Kelurahan Pulau Panggang dan Pulau Pari Kepulauan Seribu DKI Jakarta. Institut Pertanian Bogor. 2003.

[27] Turner PD, R K. Economics of National Resources and Environment. John Hopkin University Press. 1990.

[28] Prasetyo A. Pengelolaan Hutan Sistem Masyarakat. 2006.

[29] Kimmerer RW. Weaving Traditional Ecological Knowledge into Biological Education: A Call to Action. BioScience. 2002;52(5):432. doi: 10.1641/0006-3568(2002)052[0432:wtekib]2.0.co;2

[30] Robbins P, Berkes F. Sacred Ecology: Traditional Ecological Knowledge and Resource Management. Economic Geography. 2000;76(4):395. doi: 10.2307/144393

[31] Hidayat S, Umar I. Peta geologi lembar Balikpapan, Kalimantan. Pusat Penelitian dan Pengembangan Geologi. 1994.

[32] Lizarralde M. Biodiversity and loss of indigenous languages and knowledge in South America. In: Maffi L, Editor. Biocultural diversity. Smithsonian Institution Press. 2001. p. 265–80.

[33] Zarger RK, Stepp JR. Persistence of Botanical Knowledge among Tzeltal Maya Children. Current Anthropology. 2004;45(3):413–8. doi: 10.1086/420908

[34] Ellen R. Modern crises and traditional strategies. 1st ed. Berghahn Books. 2011.

[35] Padoch C. Borneo in Transition: People, Forests, Conservation, and Development. Oxford University Press. 2012.

[36] Pitcher TJ, Preikshot D. rapfish: a rapid appraisal technique to evaluate the sustainability status of fisheries. Fisheries Research. 2001;49(3):255–70. doi: 10.1016/s0165-7836(00)00205-8

