# Supplementary material for: The decline of local wisdom in managing the Wain River protected forest near Indonesia’s new capital city buffer zone
Source: PLoS One. 2025 Sep 25;20(9):e0333008. doi: 10.1371/journal.pone.0333008 (PMC12463194; doi:10.1371/journal.pone.0333008)
Supplement: S1 Appendix — (DOCX) [file pone.0333008.s002.docx]

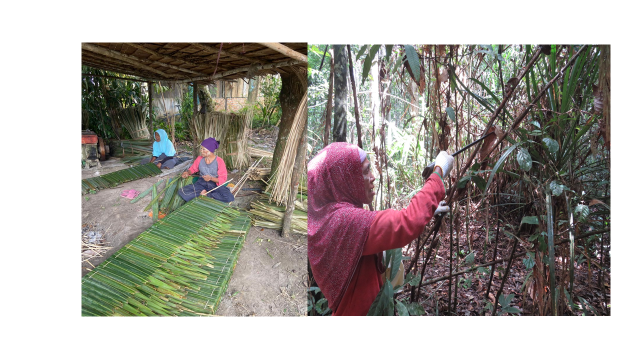


**Figure 1.** Utilizing the environmental services of the Wain River protected forest
by harvesting rattan and nipa leaves for roofing while considering sustainability
and conservation aspects. Source: Author's documentation. Published with the consent of the participant.


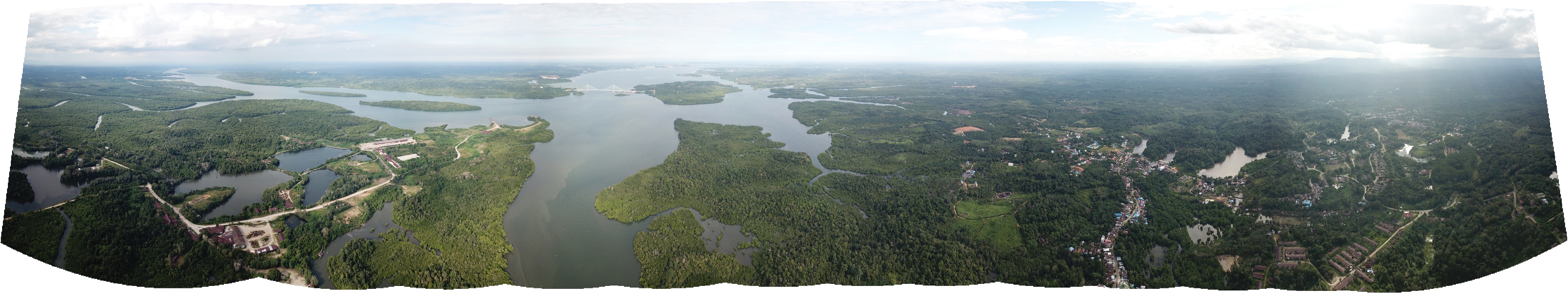


**Figure 2** Wain Protection Forest across the bay, from drone view, seen Pulau Balang Bridge across Balikpapan Bay. Source: Author's documentation.

**Figure 3.** Sustainability index value of local wisdom

The figure visualizes the ordination of local knowledge across five dimensions: economy, infrastructure-technology, socio-culture, ecology, and legal-institutional aspects. Each point represents the relative sustainability status of these dimensions, positioned along axes of local knowledge status and other distinguishing features. Reference anchors (♦) and anchors (▲) are used to contextualize the data. Source: Author’s analysis using RAP-LK method.

**Figure 4.** Attributes across sustainable dimensions: ◼ Social and Cultural Dimension ◼ Economic Dimension ◼ Environmental Dimension ◼ Legal and Institutional Dimension ◼ Technology and Infrastructure Dimension

This figure presents the relative values of sustainability-related attributes categorized into five dimensions: (1) Social and Cultural (red), (2) Economic (blue), (3) Environmental (yellow), (4) Legal and Institutional (turquoise), and (5) Technology and Infrastructure (green). Each bar represents the quantified value of a specific attribute as assessed through stakeholder input and analysis. The results highlight the strengths and gaps in each sustainability dimension, providing a comprehensive overview of local wisdom and governance capacity in forest management. Source: Author’s analysis.
